# In Silico and In Cell Analysis of Openable DNA Nanocages for miRNA Silencing

**DOI:** 10.3390/ijms21010061

**Published:** 2019-12-20

**Authors:** Sofia Raniolo, Federico Iacovelli, Valeria Unida, Alessandro Desideri, Silvia Biocca

**Affiliations:** 1 Department of Systems Medicine, University of Rome Tor Vergata, Via Montpellier 1, 00133 Rome, Italy; sofiaraniolo@gmail.com; 2 Department of Biology, University of Rome Tor Vergata, Via della Ricerca Scientifica 1, 00133 Rome, Italy; federico.iacovelli@uniroma2.it (F.I.); valeria.unida@gmail.com (V.U.)

**Keywords:** DNA nanostructure, MD simulations, DNA hairpins, stability, cell internalization, miRNA sequestering

## Abstract

A computational and experimental integrated approach was applied in order to study the effect of engineering four DNA hairpins into an octahedral truncated DNA nanocage, to obtain a nanostructure able to recognize and bind specific oligonucleotide sequences. Modeling and classical molecular dynamics simulations show that the new H4-DNA nanocage maintains a stable conformation with the closed hairpins and, when bound to complementary oligonucleotides produces an opened conformation that is even more stable due to the larger hydrogen bond number between the hairpins and the oligonucleotides. The internal volume of the open conformation is much larger than the closed one, switching from 370 to 650 nm^3^, and the predicted larger conformational change is experimentally detectable by gel electrophoresis. H4-DNA nanocages display high stability in serum, can efficiently enter the cells where they are stable and maintain the ability to bind, and sequester an intracellular-specific oligonucleotide. Moreover, H4-DNA nanocages, modified in order to recognize the oncogenic miR21, are able to seize miRNA molecules inside cells in a selective manner.

## 1. Introduction

Several materials are currently taken into consideration for building nanocarriers/nanocapsules useful in the pharmaceutical industry for therapy. Among materials, DNA is a very suitable one for several reasons. It has the intrinsic properties of excellent biocompatibility and stability, it is chemically modifiable, and it can be used to assemble nanostructures with different size and geometry [1]. It provides a safe hydrophilic environment for trapping useful payloads, and DNA motifs can be engineered to control size, shape, and switchable open/close mechanisms [2]. DNA strands can be modified with cellular recognition signals, such as folate, transferrin, or aptamers, which permit the assembly of functionalized DNA-based nanostructures (DNS) useful for selective targeting into cells through receptor-mediated mechanism [3,4,5]. Due to their intrinsic biocompatible, nontoxic, and stable properties, DNS have been extensively investigated for various biomedical applications, such as drug delivery, cellular biosensing, and *in vivo* imaging [4,5,6,7,8], and, more recently, in gene silencing and RNA anticancer therapy [9,10]. Different shape-changing structural modules can be integrated in the DNS, allowing input-induced conformational changes. For example, octahedral DNA cages have been functionalized with temperature-dependent hairpins, to allow the reversible encapsulation and release of a protein [11,12], or with pH-dependent triple helices that allow the transition from a “folded” to an “unfolded” form for the transport and release of triplex-specific binding molecules [13]. Tetrahedral DNA cages have been modified with the use of DNA oligonucleotides with pH-sensitive i-motif, to encapsulate an enzyme inside them [14]. DNA nanostructures have also been functionalized to selectively interact with intracellular miRNA, mainly to detect their concentration, using electrochemical current or fluorescence signals [15,16,17]. Here, taking advantage of our experience matured in the last years in the characterization of different types of fully covalently octahedral DNA nanocages [11,12,18,19,20,21], including their receptor-mediated cell targeting and their efficacy in selective drug delivery [5,22,23], we propose a new nanostructure for a possible therapeutic use as an efficient captor of the oncogenic miR21. For this purpose, we have initially engineered in one face of a truncated DNA cage four DNA hairpins complementary to a specific oligonucleotide (Fuel), to form a nanocage (H4-NC) with selective oligonucleotide sequestering activity. Evaluation of the structural–dynamical properties through molecular dynamics (MD) simulations indicated that the complementary oligonucleotides act as allosteric remodelers, inducing a conformational change to the H4-NC, which displays a stable opened shape that is larger than that of the closed form. H4-NC assembly, stability in biological fluids, time-dependent cellular uptake, and sequestering capability have been positively evaluated. Notably, engineering of DNA hairpins with sequence complementarity to miR21 leads to H4-nanocages with efficient miR21 sequestering activity inside cells.

## 2. Results

### 2.1. Models of the Closed/Opened States of H4 DNA Nanocage

**Figure 1:**
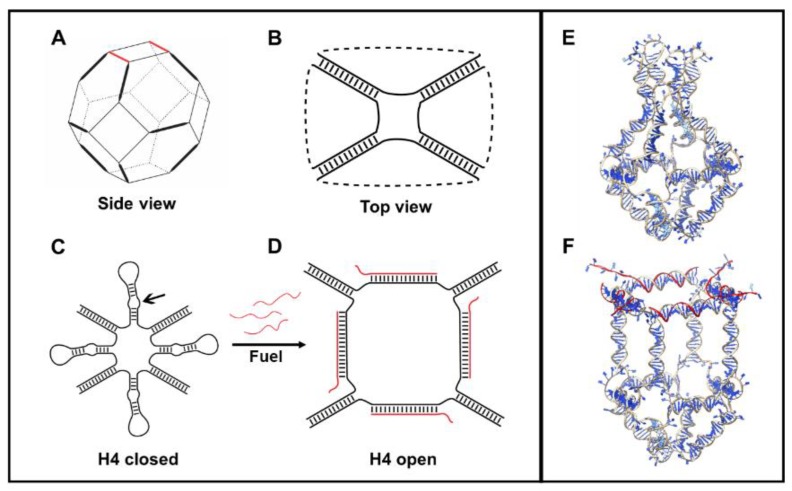
Schematic and atomistic representation of H4-NC. (**A**) DNA octahedral scaffold. (**B**) Top view of an octahedral DNA cage. (**C**) Closed representation of a H4-NC. (**D**) Opened conformation, highlighting the change upon their interaction with the allosteric remodeler (Fuel) oligonucleotides. Full atomistic representation of the closed (**E**) and opened (**F**) state of a H4-NC.

The H4 DNA nanocage (H4-NC) was designed by starting from a truncated octahedral DNA cage structure [24], composed by eight different oligonucleotides, extensively characterized by our group [11,12,18,19,20,22]. The DNA cage structure is covalently closed and composed of 12 double-stranded B-DNA helices (Figure 1A), forming the edges of the structure, connected by short single-stranded thymidine linkers constituting square truncated faces (Figure 1B). Four DNA hairpin units (H4) were introduced in one truncated face (Figure 1C), increasing the length of four of the eight oligonucleotides used for the cage assembly, to give a H4 closed cage, which is represented in Figure 1E. The hairpins are composed by a ten-base double helix connected by an 8-cytosine loop (Figure S1). Three mismatches were introduced into the hairpins (see arrow in Figure 1C), to weaken their stability and to facilitate the binding of an allosteric remodeler consisting of 35-base complementary oligonucleotide (Fuel), at the level of the loop region. Binding of the allosteric remodeler induces a conformational change in the DNA nanostructure toward an opened conformation, represented in Figure 1F and highlighted in Figure 1D, for the face containing the hairpins.

### 2.2. Computational Evaluation of the H4-DNA Nanocage Stability

**Figure 2:**
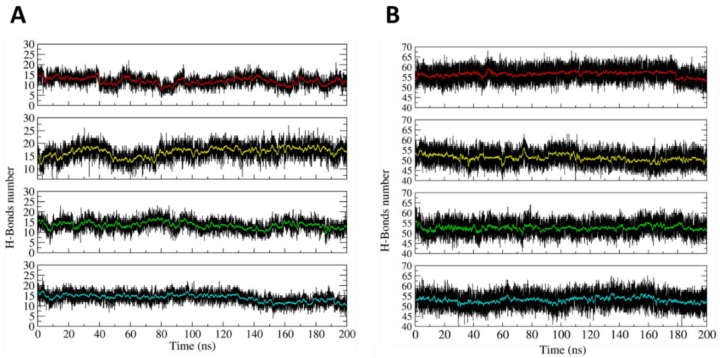
Time-dependent evolution of the hydrogen bonds (HBs) number. (**A**) Number of HBs evaluated within the four hairpins in the closed DNA nanocage. (**B**) Number of HBs between the allosteric remodelers and the hairpins on the H4 opened state. The red, yellow, green, and blue lines highlight the HBs number averaged every 100 picoseconds of simulation time.

The dynamical stability of the H4-NC in the opened and closed states was investigated at the atomistic level, using 200 ns long classical MD simulations. The main result coming from the simulation is that the two states display stable configurations, having a largely different shape. The stability of the two states can be deduced from Figure 2A,B, reporting, as a function of time, the number of hydrogen bonds (HBs) present within the hairpins in the closed and opened states, respectively. In the closed state, due to the presence of three mismatches, each hairpin maintains an average number of 15 HBs (Figure 2A). The relatively low HB number induces some local distortions, but the hairpins maintain the closed conformation, as it can be observed from a typical frame of the simulation (Figure S1). The opened state is even more stable due to the larger number of HBs between the hairpins and the allosteric remodelers. In this case, 55 is the average number of HBs in each edge of the opened H4-NC configuration (Figure 2B).

The shape of the closed and the opened states can be evaluated by plotting, as a function of time, the radius of gyration (RG) and the volume of the internal cage cavity reported in Figures S2 and S3, respectively. The RG of the opened cage (Figure S2, red line) is constantly larger than that of the closed state (Figure S2, black line), with the average RG values being 7.3 and 6.3 nm, respectively. In line, the average internal volume of the nanocage in the opened and closed state is 370 and 650 nm^3^, respectively (Figure S3). According to these values, the two conformations are predicted to display a conformational difference detectable through gel electrophoresis analysis.

### 2.3. Assembly of H4 DNA Nanocages and Their Interaction with Fuel

**Figure 3:**
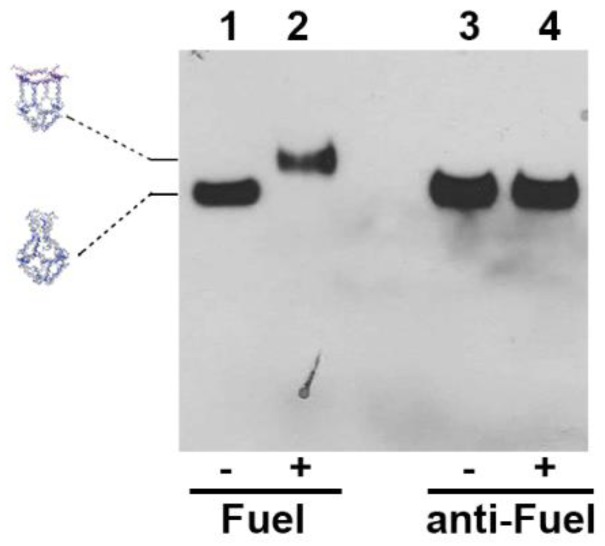
H4-NC opening reaction. DNA blot of H4-NC electrophoretic mobility in the closed and opened conformational state before (-) and after (+) incubation with 10x molar excess of Fuel and anti-Fuel oligonucleotides.

H4-NCs with truncated octahedral structures were assembled as described [22,24], by using modified oligonucleotides (Table S1), and functionalized with a biotin molecule (Bio), for their detection through the biotin–streptavidin assays. The assembly efficiency of H4-NCs was estimated to be approximately 40%, which is in agreement with previous reports of octahedral cage assembly [24]. In order to verify the opening reaction, H4-NCs were incubated with 10 times molar excess of Fuel for 30 min at 37 °C, analyzed by polyacrylamide gel electrophoresis, and visualized by GelRed nucleic acid staining (Biotium, Inc, Fremont, CA, USA) (Figure S4A) and by DNA blot, by using streptavidin-HRP (Figure 3). In the presence of Fuel, H4-NCs undergo a structural conformational change, as evidenced by a slower electrophoretic mobility corresponding to the opened form of the structure (Figure 3, lanes 1 and 2). As a control, H4-NCs were incubated with 10x molar excess of a nonrelevant oligonucleotide, characterized by the same length, but with a sequence complementary to the Fuel (anti-Fuel) (Figure 3, lanes 3 and 4). In this case, no change in electrophoretic mobility is detected, indicating that the structure remains in the closed form, due to the absence of interaction between the DNA nanocage and anti-Fuel. Titration with different Fuel:H4-NC ratios indicates 10:1 as the best ratio for obtaining a complete conformational change (Figure S4B).

### 2.4. H4 DNA Nanocages Internalization and Their Interaction with Fuel Inside Cells

**Figure 4:**
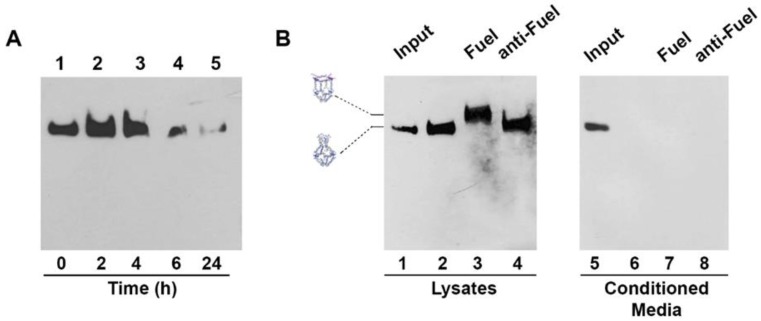
(**A**) Time-dependent uptake of H4-NCs and their stability in Hela cells. H4-NCs were internalized in the presence of transfection reagent for different times, as indicated. Lane 1 shows 40 ng of H4-NCs before incubation with cells. (**B**) H4-NCs opening reaction inside cells. HeLa cells were incubated with closed H4-NCs. After 4 h, cells were washed and incubated for 2 h, with fresh medium (lane 2) or a mixture containing transfection reagent and Fuel (lane 3) or anti-Fuel (lane 4) oligonucleotides. Lanes 6–8 show H4-NCs purified from conditioned media (CM) derived from each treatment group, as indicated. In addition, 30 ng of H4-NC before incubation with cells (input) are shown in lanes 1 and 5. H4-NCs were detected with streptavidin-HRP.

Stability of H4-NCs in biological fluids is an important preliminary step for their use in cells. We compared the stability of the closed and opened forms of H4-NCs in fetal bovine serum (FBS) at 37 °C (Figure S5). H4-NCs are stable for at least 4 h in 10% FBS, either in the closed (Figure S5, lanes 1–3) or in the opened form (Figure S5, lanes 7–10). After 4 h, they start to be degraded as a function of time. Time-dependent cellular uptake and stability of H4-NCs were investigated in HeLa cells. At the end of each incubation time with cells, H4-NCs were purified and analyzed by DNA blot, as previously described [22]. Figure 4A shows H4-NCs internalized at different times of incubation in HeLa cells. Lane 1 shows 40 ng of H4-NCs prior to incubation with cells (time 0). A large amount of H4-NCs were internalized in HeLa cells in 4 h (Figure 4A, lanes 2–3). After 6 h, a lower amount of intact H4-NCs is found in cell extracts (compare lanes 3 and 4) and, after 24 h, H4-NCs are barely detectable inside HeLa cells (lane 5). The important question to answer is whether H4-NCs, once entered in cells, interact with the Fuel and change their conformation, as shown in the test tube (Figure 3). HeLa cells were incubated with H4-NCs for 4 h, allowing for their optimal internalization, and, after extensive washing, incubated for a further 2 h with Fuel or anti-Fuel oligonucleotides mixed with the transfection reagent, allowing for their entrance, as described in Materials and Methods. H4-NCs conformational change was evaluated by DNA blot after purification of DNA structures from cells (Figure 4B). Lane 2 of Figure 4B shows that intracellular H4-NCs maintain a closed structure, identical to the control, representing 30 ng of closed H4-NCs before incubation with cells (input, lane 1). Notably, lane 3 shows that H4-NCs, purified from cells after a further 2 h incubation with Fuel and transfection agent, change their electrophoretic mobility, due to the H4-NC–Fuel interaction inside cells, leading to the opening of the structure. A titration curve of the intracellular opening reaction in the presence of different Fuel concentrations indicates that the cage-opening conformational change starts to be visible at 10x molar excess of Fuel and reaches its maximum at 30x excess (Figure S6). It is worth noting that incubation with anti-Fuel oligonucleotide (lane 4, anti-Fuel) does not result in change in the electrophoretic mobility, confirming that, also inside cells, H4-NCs open up only in the presence of the complementary Fuel oligonucleotide (Figure 4B, lane 3). The analysis of conditioned media (CM) derived from the internalization experiment shows that no bands are visible in the CM (Figure 4B, lanes 6–8), indicating that the large majority of H4-NCs were internalized by cells.

### 2.5. H4-DNA Nanocages for miRNA 21 Sequestering: In Vitro and In Cell Selective Interaction

**Figure 5:**
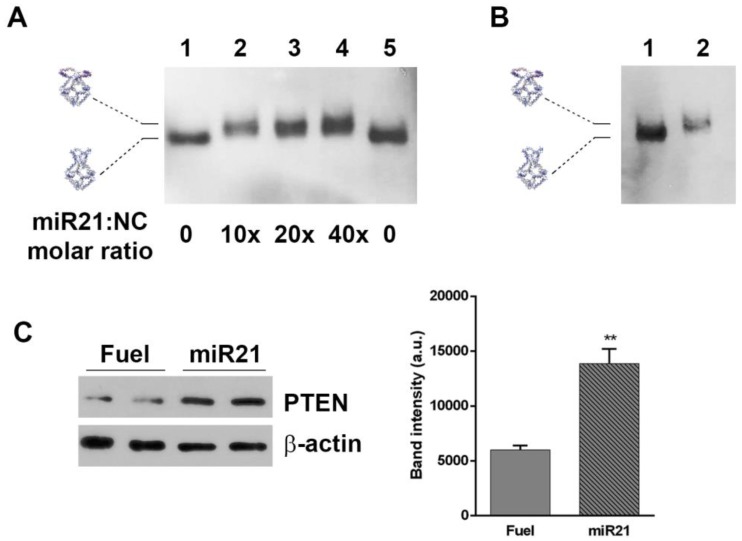
H4-miR21-NC opening reaction *in vitro* (**A**), miR21 sequestering activity in HeLa cells (**B**), and their effect on PTEN target gene (**C**). (**A**) DNA blot of miR21-NCs in the closed (lanes 1 and 5) and opened (lanes 2–4) conformational state after incubation with miR21 at different molar excess. (**B**) Lane 2 shows H4-miR21-NC incubated for 4 h in HeLa cells in the presence of transfection reagent, purified and analyzed by DNA blot. Lanes 1 shows 30 ng of H4-miR21-NCs before incubation with cells. (**C**) Western blotting on cell lysates from Hela cells treated with H4-miR21-NCs as compared to H4-Fuel-NCs on miR21 target gene PTEN is shown. β-actin was used as internal control. Densitometric analysis of three different experiments of each experimental condition is shown on the right panel. (**) *p* < 0.01.

One application of H4-NCs is their use for sequestering intracellular small RNAs, such as miRNAs. The four hairpins forming oligonucleotides were engineered with sequences complementary to miR21 (Table S1), which is overexpressed in various tumors and cell lines, including HeLa cells, and is considered to be an excellent target for therapeutic intervention. Figure 5 shows the *in vitro* opening reaction of the H4-miR21-NC, incubating them with different miR21 molar excess for 30 min at 37 °C and analyzed by DNA blot. The titration curve shows that H4-miR21-NC, in the presence of miR21, has a slower electrophoretic mobility, indicative of a miR21-nanocage interaction (Figure 5A, lanes 2–4). For testing the miRNA sequestering activity of miR21 with NCs in cells, HeLa cells were incubated for 4 h, with H4-miR21-NCs, in the presence of transfectant, to allow their internalization. At the end of the incubation time, nanocages were purified and analyzed by DNA blot. Figure 5B shows that they run with a slightly lower electrophoretic mobility compared to closed H4-NCs not incubated with cells (compare lanes 1 and 2), indicating that internalized H4-miR21-NCs are able to bind the intracellular miR21 and undergo the same conformational change observed in the *in vitro* experiment (Figure 5A).

To determine the biological effect of H4-miR21-NCs, we studied the expression level of PTEN, a known downstream gene regulated by miR21 [25]. Figure 5C shows the PTEN expression level analyzed by Western blot of extracts derived from H4-miR21-NC-treated HeLa cells. Densitometric analysis is shown in the right panel of Figure 5C. In detail, 24 h after transfection, PTEN expression level was 2.4 times higher in H4-miR21-NC treated versus H4-Fuel-NC treated negative controls, indicating a true knockdown of miR21 by DNA nanocages.

## 3. Discussion

We have engineered a DNA nanostructure by introducing, into one face of an octahedral nanocage, four DNA hairpins complementary to an oligonucleotide sequence, obtaining a new nanostructure, called H4-NC, which is able to seize four copies of a specific oligonucleotide. MD simulations show that the H4-NC displays two stable conformations, a closed and an open state, and that the opened structure has a volume almost twice as large as the closed one. The high thermodynamic stability is coupled to a high biological stability. In fact, H4-NCs are stable both in serum and inside cells, similarly to other previously characterized covalently linked nanostructures of different size and geometry, although with a lower stability when compared to the unmodified octahedral cages [5,22]. These new cages can be efficiently uptaken by the cells, and, importantly, they undergo the Fuel-binding-induced conformational change inside cells, suggesting that they can be used for therapeutic purposes, such as captors of specific oncogenic miRNA. Indeed, our results demonstrate that engineering the four hairpins forming oligonucleotides with sequences complementary to miR21 gives rise to a nanocage which can efficiently sequester miR21, both *in vitro* and *in cells*. After internalization in HeLa cells, H4-miR21-NCs run with a slightly lower electrophoretic mobility compared to H4-miR21-NCs not incubated with cells (Figure 5B), indicating a direct binding of NCs to the intracellular miR21. Upon confirmation of NCs entering into cells and maintaining their target binding activity, experiments were expanded to test their use as therapeutic nanoparticles. It has been reported that overexpression of the onco-miR21 leads to the downregulation of tumor-suppressor genes, such as PTEN [25]. Here, we show that specific nanocages harboring the anti-miR21 hairpins, developed to target endogenous miR21, have a strong effect on upregulating PTEN expression when delivered to cells, confirming the efficient H4-miR21-NCs silencing activity. This result indicates that the here-described H4-NCs can be applied as therapeutic nanoparticles, either for release of a useful payload or for short RNA sequestering. In line, a minimal DNA cage was designed for encapsulation of small RNAs and conditional release in the presence of selected trigger strands [26], and DNA nanotubes, carrying multiple DNA segments, were proposed for capturing overexpressed oncogenic miRNAs [9]. DNA-based nanostructures, mainly based on a tetrahedral geometry, were implemented for miRNA biosensing, using electrochemical, optical, and microscopic strategies as sensing approaches [17]. For example, tetrahedral DNS immobilized on the surface of a gold electrode have been proposed for electrochemical sensing of microRNAs, showing enhanced accessibility to the target when compared to linear single-stranded probes [27]. Tetrahedra have also been used for miRNA detection, exploiting the optical properties of a fluorophore and a quencher [15]. Here we expand the use of truncated DNA octahedra, extensively characterized by us [19,23,24] as miRNA sequestering machines. These structures, modifiable on each truncated square face through the insertion of sequences complementary to different miRNAs, represent a ductile framework to act as multiple miRNAs sequestering units and restore the basal level of overexpressed oncomiRs in cancer cells.

## 4. Materials and Methods

### 4.1. H4-Nanocage Modeling and Molecular Dynamics Protocol

The scaffold of the octahedral DNA nanocage was built with Polygen software (https://portal.if.usp.br/gfcx/pt-br/node/345, accessed on 12/10/2019) [20], using oligonucleotides sequences previously designed to experimentally assemble truncated octahedral geometries [24]. The sequences of the DNA hairpins and of the oligonucleotides acting as allosteric remodelers were designed and optimized through a thermodynamic approach, using NUPACK (http://nupack.org/, accessed on 12/10/2019) [28]. The sequences, reported in Supplementary Materials (Table S1), were progressively altered by introducing mismatches into the double helical portion of the hairpin, to increase the stability of the remodeler–hairpin complex (Figure S1). The H4-NCs were generated with SYBYL 6.0 program (CERTARA, http://www.certara.com, accessed on 12/10/2019), manually adding to the octahedral structure four DNA hairpins, modeling the closed (Figure 1C, left) and opened (Figure 1C, right) states. The steric clashes introduced by the modeling procedure were removed through the SYBYL anneal module, followed by a minimization of the entire structure, using the SYBYL maximin2 module. The system topologies and the coordinates, used as input for the NAMD 2.12 MD package (Champaign, IL, USA) [29], were obtained through the AmberTools16 tLeap module (San Francisco, CA, USA) [30], parameterizing the structures with the AMBER parmbsc1 force field [31]. The structures were inserted into a cubic box filled with TIP3P water molecules [32], imposing a minimum distance between the cage and the box of 14 Å. The charges were neutralized by adding, in electrostatically favorable positions, magnesium counter-ions to the solvated systems. The modeled cage was subjected to two minimization runs. In the first one, restraints of 5.0 kcal/mol·Å^−2^ were imposed on all the cage atoms, to relax the water molecules and the ions; in the second one, the cage was minimized without any restraint, to relax the entire system. The minimized structure was thermalized, in the NVT ensemble, increasing the temperature of 10 K every 30 ps from 0 to 300 K. The optimized systems were then simulated by using periodic boundary conditions for 200 ns, with a 2.0 fs time-step, using the isobaric–isothermal ensemble (NPT). The electrostatic interactions were calculated every 4.0 fs, using a cut-off of 10 Å for the evaluation of short-range nonbonded interactions and the PME method [33] for the long-range electrostatic interactions. The SETTLE [34] algorithm was used to constrain the nucleic acids and the water molecules. Temperature was fixed at 300 K, using Langevin dynamics [35], while pressure was held constant at 1 atm through the Langevin piston method [36]. The atomic positions were saved every 1000 steps (2.0 ps), for the analyses. The simulations were performed by using 10 nodes, for a total of 480 CPUs, on the CRESCO6 partition of the ENEA HPC cluster [37].

### 4.2. Trajectory Analysis

Gyration radius was calculated by using the gmx gyrate of the Gromacs 2018.1 package (Stockholm, Sweden) [38], whereas the hydrogen bond number was evaluated, through the gmx hbond module, using an angle cut-off (hydrogen-donor-acceptor) of 30° and a maximum donor-acceptor distance of 3.5 Å. The internal volume of H4 cages was calculated by using the program EPOCK 1.0.5 (Paris, France) [39]. Pictures were obtained by using the UCSF Chimera 1.13 program (San Francisco, CA, USA) [40], while graphs were produced by using the Grace plotting tool (http://plasma-gate.weizmann.ac.il/Grace/, accessed on 12/10/2019).

### 4.3. Preparation of Octahedral H4-Nanocages

H4-NCs were prepared as described for octahedral DNA nanocages [22]. Oligonucleotides used for the assembly of H4-NCs were HPLC-purified and the sequences are reported in Table S1.

### 4.4. Stability of H4-Nanocages

Biotinylated H4-NCs were incubated in 10% fetal bovine serum (FBS) (Gibco, Paisleg, UK), at 37 °C for different times. After incubation, each sample was treated with proteinase K (Promega, WI, USA)(100 μg/mL) for 1 h at 37 °C, and protein digestion was stopped by adding phenylmethylsulfonyl fluoride (PMSF) (Euroclone, Devon, UK), to a final concentration of 5 mM. Samples were mixed with loading buffer (Tris-Cl 500 mM, pH 6.8; glycerol 20%; SDS 4%; bromophenol blue 0.02%) and analyzed by DNA blot, as previously described [22].

### 4.5. Cell Cultures and Transfection

HeLa cells, derived from human cervix cancer, were grown in DMEM (Dulbecco’s modified Eagle’s medium) (Biowest, Miami, FL, USA) supplemented with 10% FBS (Gibco, Paisleg, UK), 1mM of L-glutamine (Sigma Aldrich, St Louis, MO, USA), 1mM sodium of pyruvate (Biowest, Miami, FL, USA), and 100 U/mL of penicillin––streptomycin (Euroclone, Devon, UK). JetPEI transfection reagent (Polyplus Transfection, Illkirch, France) was used, following the manufacturers’ protocol.

### 4.6. Purification of H4-Nanocages and DNA Blot

Cells were plated in 48-well plates at a density of 3 × 10^4^ cells/well and grown 24 h before the experiment. Cells, incubated with H4-NCs at different concentrations and times (as indicated in each experiment) were then lysed, centrifuged, digested with proteinase K, and analyzed by DNA blot, as previously described [22]. Biotin detection was carried out, using streptavidin-HRP (Horseradish Peroxidase) (Abcam Inc, Toronto, ON, Canada), and visualized by enhanced chemiluminescence (ECL Extend, Euroclone, Devon UK). For purification of H4-NCs from conditioned medium, medium was collected from each well, cleared from cellular debris by centrifugation at 10,000 rpm for 15 min, digested with proteinase K, and analyzed by DNA blot [22]. Input samples of H4-NCs are DNA structures added to cell-culture medium and immediately digested with proteinase K and processed for DNA blot.

### 4.7. Western Blot

After incubation with NCs, HeLa cells were lysed in ice-cold extraction buffer containing 10 mM of Tris/HCl (pH 7.6), 100 mM of NaCl, 10 mM of EDTA, 0.5% Nonidet P-40, 0.5% sodium deoxycholate, protease inhibitor cocktail set III (Sigma Aldrich, St. Louis, MO, USA), and 1 mM of PMSF and centrifuged for 20 min at 4 °C at 12,000 rpm. The protein concentration was determined by using Bradford assay (Sigma-Aldrich, St. Louis, MO, USA). The supernatant fraction was analyzed by SDS-polyacrylamide gel electrophoresis in 10% acrylamide gels and transferred to polyvinylidene difluoride membranes (Amersham Biosciences, Bath, UK) for 30 min at 15 V (Trans Blot Turbo Bio-Rad Laboratories, Hercules, CA, USA). PTEN rabbit mAb (Cell Signaling Technologies, Danvers, MA, USA) at dilution 1:1000 and β-actin mouse mAb (Cell Signaling Technologies, Danvers, MA, USA) at dilution 1:1000 were used as primary antibodies. HRP-conjugated AffiniPure donkey anti-mouse IgG and HRP-conjugated AffiniPure donkey anti-rabbit IgG secondary antibody (Jackson Immunoresearch, Cambridgeshire, UK) at dilution 1:10,000 were used as secondary antibodies. Immunoreactive bands were visualized by enhanced chemiluminescence (ECL Extend, Euroclone, Devon, UK). The ECL data were scanned and analyzed by ImageJ software. Band intensities were normalized to β-actin. Data were analyzed by using Student’s *t*-test. Results are expressed as a mean ± SEM, calculated by using GraphPad Prism. Differences were considered statistically significant when *p* < 0.01 (**).

## Supplementary Materials

Supplementary materials can be found at https://www.mdpi.com/1422-0067/21/1/61/s1.

## Abbreviations

DNS	DNA-based nanostructures
NC	Nanocage
H4	Four-hairpin
CM	Conditioned media
MD	Molecular dynamics
RG	Gyration radius
HB	Hydrogen bonds
