# Supplementary material for: In Silico and In Cell Analysis of Openable DNA Nanocages for miRNA Silencing
Source: Int J Mol Sci. 2019 Dec 20;21(1):61. doi: 10.3390/ijms21010061 (PMC6981788; doi:10.3390/ijms21010061)
Supplement: Supplementary file 1 [file ijms-21-00061-s001.pdf]

International Journal of
Molecular Sciences

MDPI

Supplementary Materials

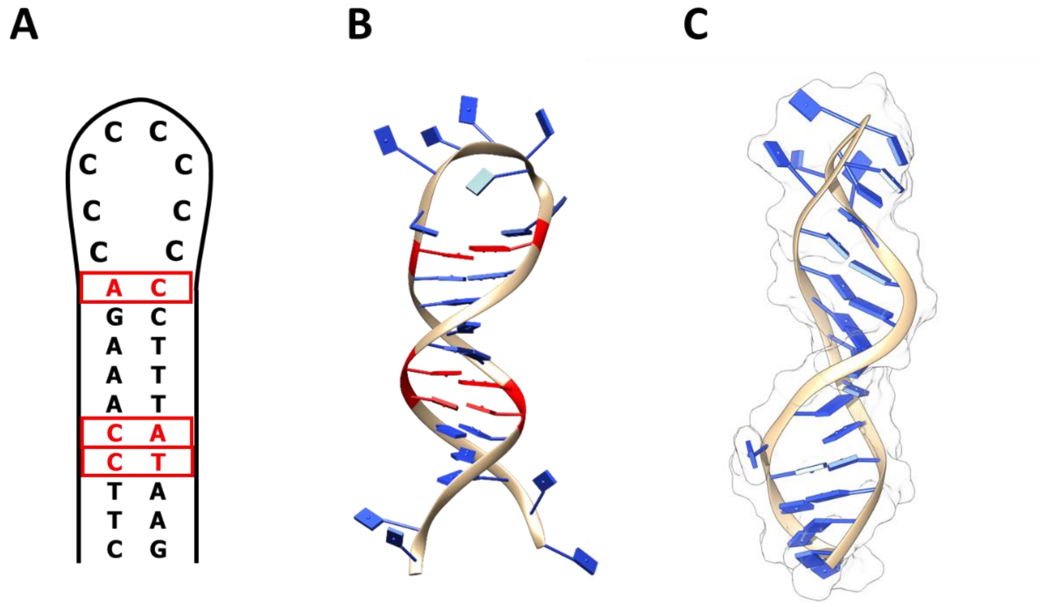

Figure S1. Schematic and atomistic representation of a single DNA hairpin. (**A**) Schematic and sequence representation of the DNA hairpin. The red boxes indicate the three mismatches. (**B**) Atomistic representations of the DNA hairpin. Mismatching bases are colored in red. (**C**) Average structure of the DNA hairpins hosted in the cage extracted from the MD trajectory. The structure is distorted but maintains almost all the starting HBs interactions.

International Journal of
Molecular Sciences

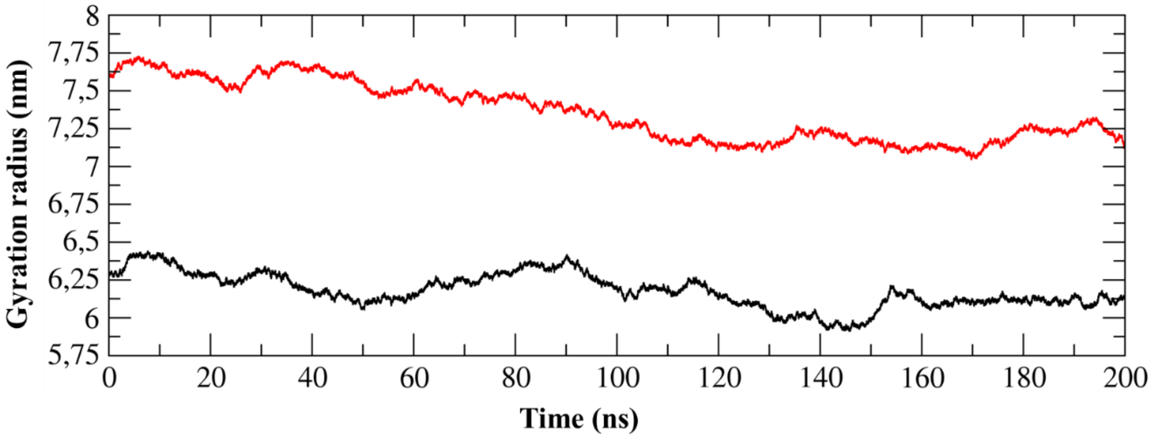

Figure S2. Time dependent evolution of the DNA nanocages gyration radius. Time-dependent evolution of the nanocages gyration radius, calculated for the closed (black line) and open (red line) states, respectively.

International Journal of
Molecular Sciences

MDPI

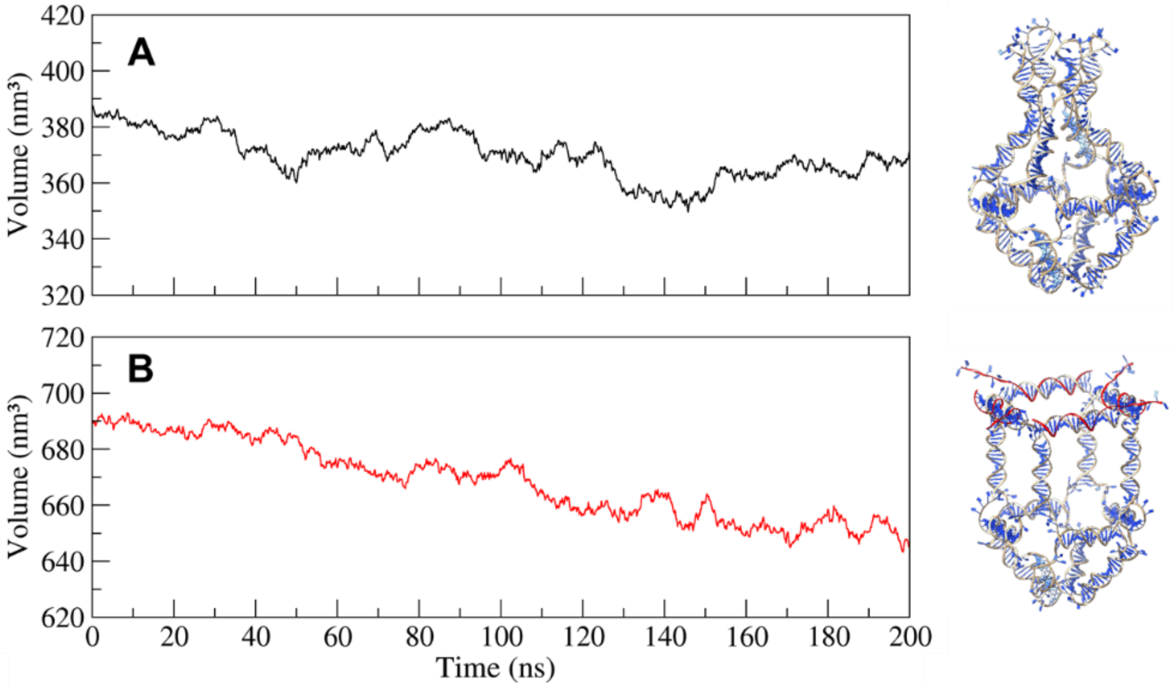

Figure S3. Time dependent evolution of the DNA nanocages internal volumes. Time dependent evolution of the structure internal cavity volume. Internal volume of (**A**) closed and (**B**) opened H4-NCs.

International Journal of Molecular Sciences

MDPI

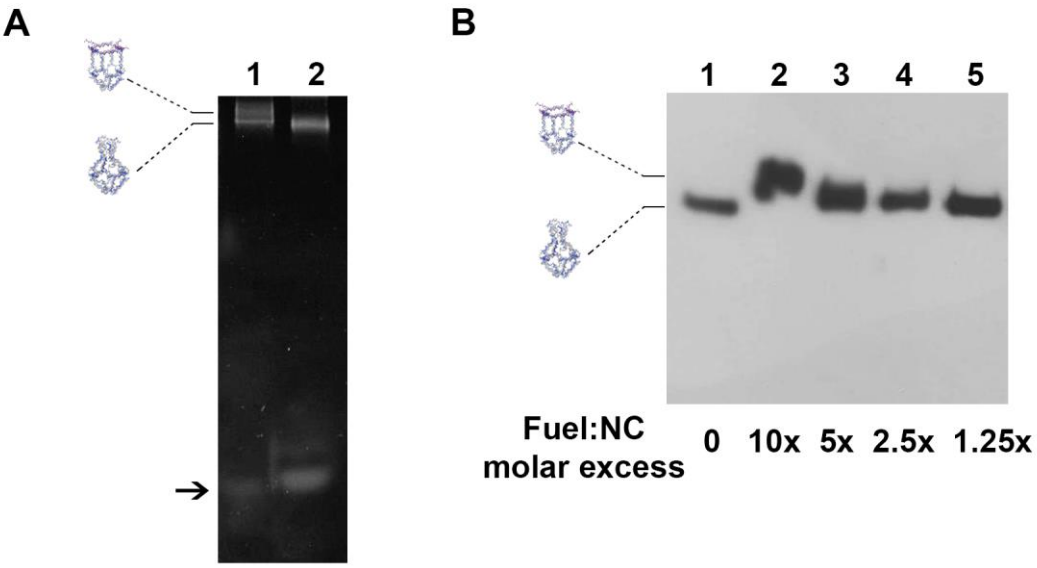

Figure S4. Titration of Fuel-nanocages opening reaction. (**A**) Polyacrylamide gel electrophoresis analysis of H4-NCs incubated with Fuel (lane 1) and anti-Fuel (lane 2). Arrow indicates the oligonucleotide bands. Notably, the intensity of the band corresponding to Fuel in lane 1 is much lower than the anti-Fuel band in lane 2, indicating that a consistent amount of the oligonucleotide Fuel is bound to H4-NCs. (**B**) Representative DNA blot analysis of H4-NC incubated with different concentration of Fuel oligo. Fuel:NC molar ratios are indicated under the gel. In order to assess the Fuel:NC molar ratio required to induce the open state, H4 DNA nanocages (H4-NC) were incubated with increasing concentration of Fuel oligonucleotide, ranging from 1.25 to 10 times molar excess, for 30 min at 37 °C. After incubation with Fuel, H4-NCs were analysed by DNA blot and visualized by using streptavidin-HRP. In panel B, lane 1 shows the electrophoretic mobility of 40 ng of closed H4-NC in the absence of Fuel. Lanes 2 to 5 show the variation of the electrophoretic mobility of the H4-NC in the presence of different concentration of oligo Fuel. At 10x Fuel molar excess, H4-NCs undergo a complete structural conformational change, as evidenced by a slower electrophoretic mobility (lane 2). Changes in the electrophoretic mobility of the H4-NC start to be visible with 5x Fuel molar excess (lane 3).

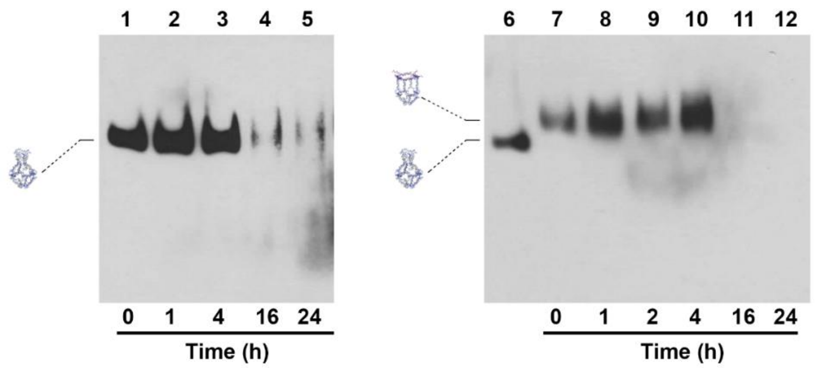

Figure S5. Stability of closed and opened H4-NCs in serum. DNA blot analysis of closed (lanes 1–5) and opened H4-NCs (lanes 6–12) incubated with 10% FBS for different times and detected by using Streptavidin-HRP. For testing the stability of the opened state, DNA nanocages were incubated in the presence of 10x molar excess of Fuel for 30 minutes to switch their conformation. Closed and opened structures were incubated with 10% FBS for different time intervals at 37 °C, digested with Proteinase K (100 μg/mL), run in 5% polyacrylamide gel and blotted to examine their structural integrity. Lanes 1 and 6 represent the closed form of H4-NCs and lane 7 the opened one, before the incubation with 10% FBS (time 0). H4-NCs are stable for at least 4h in 10% FBS at 37 °C either in the closed (lanes 1–3) or in the opened form (lanes 7–10). After 4 h, they start to be degraded as a function of time. Closed H4-NCs are more stable than the opened ones, being still detectable after 24 h of incubation (lane 5), while the opened H4-NCs are totally degraded after 16h (lane 11).

International Journal of Molecular Sciences

MDPI

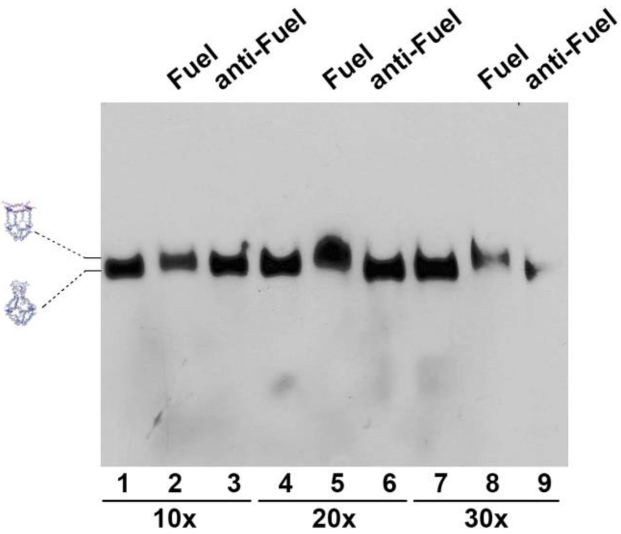

Figure S6. Titration of H4-NCs opening in cells. H4-NC conformational change after purification from cell lysates. HeLa cells were transfected for 4 h at 37 °C with closed H4-NCs at concentration of 1 μg/mL (corresponding to 5 nM). After transfection, cells were thoroughly washed with PBS and incubated for a further 2 h with a mixture containing JetPEI transfection reagent (Polyplus Transfection, Illkirch, France) and different concentration (10×, 20× and 30× molar excess) of Fuel or anti-Fuel or fresh medium as controls. H4-NC conformational change inside HeLa cells was checked by DNA blot after purification of DNA structures from cells. H4-NCs incubated with Fuel-transfection mixture change their electrophoretic mobility indicating that DNA nanocages undergo a conformational change and open up after the recognition and binding of the Fuel oligonucleotides (lanes 2, 5 and 8, Fuel). Of note, the conformational change starts to be visible when 10X molar excess of Fuel is used (lane 2) and become more evident with 30X molar excess (lane 8). Incubation with the medium (lanes 1, 4 and 7) or with a mixture containing transfection reagent and anti-fuel oligonucleotides (lanes 3, 6 and 9, anti-Fuel) does not change the electrophoretic mobility of the input, confirming that they remain in the closed state.

International Journal of
Molecular Sciences

Oligo	Sequences (5′–3′)
OL1	GCCACCAGGTTTTTCGATGTCTAAGCTGACCGTCGAATATTTCCCCCCCCCCAGAAACCTTCCTGGACCGTGATTCCATGACTTTTTCTTAGAGTT
OL2	TGGCTACAGTCGAATATTTCCCCCCCCCCAGAAACCTTCCTCGGTCAGCTTAGACATCGTTTTTGAACTCTATGCTCGGACGTTTTTGGCTCACAT
OL3	TCACGGTCCAATATTTCCCCCCCCCCAGAAACCTTCCTCTATCCGATCGAGGCATGTTTTTCATACTGAGAGCGTTCCGTTTTTGTCATGGAA
OL4	CAGATACGCTTTTTCATGCCTCGATCGGATAGTCGAATATTTCCCCCCCCCCAGAAACCTTCCTCTGTAGCCAATGTGAGCCTTTTTGTCGCAGTT
OL5	CTCAGTATGTTTTTCGGTTACGGTACAATGCCTTTTTGCCAAGACGTTAGTGTCCTTTTTCGGAACGCT
$OL6_{BIO}$	GGTGTATCGTTTTTGGCATTGTACCGTAACCGTTTTTGCGTATCTGAACTGCGACTTTTTCCACCGAAT
OL7	CGTCTTGCGTTTTTGTATGACGCAGCACTTGCTTTTTCCTGGTGGCAACTCTAAGTTTTTGGACACTAA
OL8	ATAGGATTCTTTTTGCAAGTGCTGCGTCATACTTTTTCGATACACCATTCGGTGGTTTTTCGTCCGAGC
Fuel	GAAGGTTTCGGGGGGGGGGAAATATTCAAAAAAA
anti-Fuel	TTTTTTTGAATATTTCCCCCCCCCCAGAAACCTTC
OL_{1miR}	GCCACCAGGTTTTTCGATGTCTAAGCTGACCGTCTTTCCTTTTTCAACATCAGTCTGATAAGCTATTTCCTTTTCTGGACCGTGATTCCATGACTTTTTCTTAGAGTT
OL_{2miR}	TGGCTACAGTCTTTCCTTTTTCAACATCAGTCTGATAAGCTATTTCCTTTTCTCGGTCAGCTTAGACATCGTTTTTGAATCCTATGCTCGGACGTTTTTGGCTCACAT
OL_{3miR}	TCACGGTCCTCTTTCCTTTTTCAACATCAGTCTGATAAGCTATTTCCTTTTCTCTATCCGATCGAGGCATGTTTTTCATACTGAGAGCGTTCCGTTTTTGTCATGGAA
OL_{4miR}	CAGATACGCTTTTTCATGCCTCGATCGGATAGTCTTTCCTTTTTCAACATCAGTCTGATAAGCTATTTCCTTTTCTCTGTAGCCAATGTGAGCCTTTTTGTCGCAGTT
miR21	MeOU-MeOA-MeOG-MeOC-MeOU-MeOU-MeOA-MeOU-MeOC-MeOA-MeOG-MeOA-MeOC-MeOU-MeOG-MeOA-MeOU-MeOG-MeOU-MeOU-MeOG-MeOA

Table S1. Oligonucleotide sequences. All oligonucleotides were HPLC purified and purchased from Sigma Aldrich (St. Louis, Mo, USA) (Fuel and anti-Fuel), Integrated DNA Technologies (Coralville, IA, USA) (OL_5, OL_7 and OL_8) and LGC Biosearch Technologies (Risskov, DK) (OL_1, OL_2, OL_3, OL_4, OL_{6BIO}). The sequences of the oligonucleotides are reported in Table S1. The 5′ of each oligonucleotide is phosphorylated. TTTTT represents a short non-pairing spacer inserted within the strands as a DNA junction at each vertex of the assembled 3D structure. $OL6_{BIO}$ has a biotin tetra-ethylene-glycol molecule (BtndT) at the T represented in red.
